# Thank you to the *Journal of the Medical Library Association* reviewers in 2024

**DOI:** 10.5195/jmla.2025.2192

**Published:** 2025-04-18

**Authors:** Jill T. Boruff, Michelle Kraft, Alexander J. Carroll

**Affiliations:** 1 jill.boruff@mcgill.ca, Co-Lead Editor, *Journal of the Medical Library Association*, Associate Librarian, Schulich Library of Physical Sciences, Life Sciences, and Engineering, McGill University, Montreal, QC, Canada; 2 kraftm@ccf.org, Co-Lead Editor, *Journal of the Medical Library Association*, Medical Library Director, Cleveland Clinic Foundation, Cleveland, OH, United States; 3 alexander.j.carroll@vanderbilt.edu, Associate Editor, *Journal of the Medical Library Association*, Associate Director, Stevenson Science and Engineering Library, Vanderbilt University, Nashville, TN, United States

## Abstract

We sincerely thank the 145 peer reviewers in 2024 who helped evaluate and improve the quality of work published in the *Journal of the Medical Library Association (JMLA)*. We are always looking to expand our pool of reviewers who can critically comment on any topic of research or practice in health sciences librarianship. If you are interested in serving as a peer reviewer for *JMLA*, please express your interest to the editors at jmla@journals.pitt.edu.

## PEER REVIEWERS WHO SERVED

Nancy Adams

Samuel Adeosun

Zarif Bin Akhtar

Karen S. Alcorn

Alexandre Amar-Zifkin

Alice Anderson

Christine Andresen

Catherine Arnott Smith

Elizabeth Ayorinde

Paul Bain

Becky Baltich Nelson

Stacy Brody

Roy Eugene Brown

Amelia Brunskill

Nicole Capdarest-Arest

Tallie Casucci

Robin E Champieux

Liza Chan

Alain Manuel Chaple-Gil

Yin Yin Chen

Katherine Chew

Marina Chilov

Merete Christianson

Jamie Conklin

Jill Crawley-Low

John W. Cyrus

Jill Daby

Jennifer Davis

Sandy De Groote

Basia Delawska-Elliott

Liz Dennett

Patricia Devine

Deborah Divis

Margaret Easbey

Jonathan Eldredge

Amy Faltinek

Lisa Federer

Bethany Figg

Jensen Fisher

Mary Grace Flaherty

David Flynn

Elizabeth Frakes

Helen Fulbright

Heather Ganshorn

Gina Genova

Dean Giustini

Julie Glanville

Adelia Bush Grabowsky

Alyssa Grimshaw

Rosie Hanneke

Eli Harriss

Andrea Harrow

Sike He

Andy Hickner

Rachel J. Hinrichs

Toni Hoberecht

Anna Hughes

Emily Hurst

Natalie Hutchinson

Emily M. Johnson-Barlow

Shannon D. Jones

Kellie Kaneshiro

Christiana Keinath

Megan Kennedy

Chana Kraus-Friedberg

Evan Kuehn

Janice Yu Chen Kung

Erica Lake

Leena Lalwani

Haoyong Lan

Janna C. Lawrence

Paul Levay

Anne M. Linton

Laura Lipke

Diane L. Lorenzetti

Ya-Ling Lu

cuncun1 lu

Tara Malone

Derek Hunter Marshall

Sarah McClung

Zahra Mehri

Laura Menard

Misa Mi, AHIP

Jolene M. Miller

Elizabeth Moreton

Anna Beth Morgan

Rebecca Morin

Martin Morris

Patricia Morton

Annie Nickum

Matthew Noe

David Nolfi

Hannah F. Norton

Robin MN Parker

Gerald Perry

Eleni Philippopoulos

Brandon Pieczko

Dawid Pieper

Caitlin Plovnik

Stacy Posillico

Carrie Price

Behrooz Rasuli

Kevin Read

Jason B. Reed

Rebecca Reznik-Zellen

Alisa Rod

Ezzie Rodgers

Guillermo Armando Ronda-Pupo

Amanda Ross-White

Ellen Rubenstein

Christopher Ryland

Devin Savage

Julie Schiavo

Hannah Schilperoort

Cynthia Marie Schmidt

Jan W. Schoones

Stephanie J. Schulte

April J. Schweikhard

Jessica Sender

Carol Shannon

Tracy C. Shields

Anita Slack

Judy Smith

Daniela Solomon

Amanda Damasceno de Souza

Priscilla Stephenson

Julia Stumpff

Alisa Surkis

Natalie Tagge

Nedelina Tchangalova

Angela Tucker

Shelie Vacek

Kathryn Vanderboll

Marc von Gernler

Roland Bernard Welmaker

Monique Wessels

Rachel Whitney

Erik Wikström

Gloria Willson

Alexandria Quesenberry Wilson

Michelle Yee

Semhar Yohannes

Jonathan Young

Emily Zimmermann

Sarah Wade

